# Timing Matters: Effects of Early and Late Estrogen Replacement Therapy on Glucose Metabolism and Vascular Reactivity in Ovariectomized Aged Wistar Rats

**DOI:** 10.1155/2023/6683989

**Published:** 2023-11-15

**Authors:** Diana Ramírez-Hernández, Pedro López-Sánchez, Diego Lezama-Martínez, Neidy M. Kuyoc-Arroyo, Jessica E. Rodríguez-Rodríguez, Salvador Fonseca-Coronado, Ignacio Valencia-Hernández, Jazmin Flores-Monroy

**Affiliations:** ^1^Myocardial Pharmacology Laboratory, Faculty of Higher Studies Cuautitlan, National Autonomous University of Mexico, 54740 State of Mexico, Mexico; ^2^Laboratorio de Farmacología Molecular, Escuela Superior de Medicina, Instituto Politécnico Nacional, 11340 Ciudad de México, Mexico; ^3^Biological Pharmaceutical Chemist Career, Faculty of Higher Education Zaragoza, National Autonomous University of Mexico, Batalla 5 de Mayo S/N, Ejército de Oriente, Iztapalapa, 09230 Mexico City, Mexico; ^4^Laboratory 7, Biomedicine Unit, Faculty of Higher Education Iztacala, National Autonomous University of Mexico, Avenida de los Barrios 1, Los Reyes Ixtacala, 54090 Tlalnepantla de Baz, Mexico; ^5^Immunology Laboratory, Faculty of Higher Studies Cuautitlan, National Autonomous University of Mexico, 54740 State of Mexico, Mexico; ^6^Laboratorio de Farmacología Cardiovascular, Escuela Superior de Medicina, Instituto Politécnico Nacional, 11340 Ciudad de México, Mexico

## Abstract

Cardiovascular disease incidence increases after menopause due to the loss of estrogen cardioprotective effects. However, there are conflicting data regarding the timing of estrogen therapy (ERT) and its effect on vascular dysfunction associated with impaired glucose metabolism. The aim of this work was to evaluate the effect of early and late ERT on blood glucose/insulin balance and vascular reactivity in aged ovariectomized Wistar rats. Eighteen-month-old female Wistar rats were randomized as follows: (1) sham, (2) 10-week postovariectomy (10 w), (3) 10 w postovariectomy+early estradiol therapy (10 w-early E2), (4) 20-week postovariectomy (20 w), and (5) 20-week postovariectomy+late estradiol therapy (20 w-late E2). Early E2 was administered 3 days after ovariectomy and late therapy after 10 weeks, in both groups. 17*β*-Estradiol (E2) was administered daily for 10 weeks (5 *μ*g/kg/day). Concentration-response curves to angiotensin II, KCl, and acetylcholine (ACh) were performed. Heart rate (HR), diastolic and systolic blood pressure (DBP and SBP), glucose, insulin, HOMA-IR, and nitric oxide (NO) levels were determined. Higher glucose levels were found in all groups compared to the sham group, except the 20 w-late E2 group. Insulin was increased in all ovariectomized groups compared to sham. The HOMA-IR index showed insulin resistance in all ovariectomized groups, except for the 10 w-early E2 group. The 10 w-early E2 group increased NO levels vs. the 10 w group. After 10 w postovariectomy, the vascular response to KCl and Ach increases, despite early E2 administration. Early and late E2 treatment decreased vascular reactivity to Ang II. At 20-week postovariectomy, DBP increased, even with E2 administration, while SBP and HR remained unchanged. The effects of E2 therapy on blood glucose/insulin balance and vascular reactivity depend on the timing of therapy. Early ERT may provide some protective effects on insulin resistance and vascular function, whereas late ERT may not have the same benefits.

## 1. Introduction

Cardiovascular disease (CVD) is the leading cause of death in women. Since 1994, the annual CVD mortality rate for women has remained higher than that for men. However, over the past decade, there has been a marked reduction in CVD mortality in women [1], probably because of increased awareness of the disparities in disease pathophysiology between the sexes, including identification of CVD risks; that is, women have a greater risk of developing the vascular coronary disease, endothelial dysfunction, and heart failure with preserved ejection fraction; meanwhile, men often present cardiac disorders and heart failure with reduced ejection fraction [2–4]. In addition, the onset of the climacteric and menopause in women is an important factor in the development of CVD. Within the first year after the last menstrual period, known as early menopause, it is when more metabolic changes occur [5].

One of the major metabolic changes during menopause is insulin resistance, which leads to the development of diabetes mellitus [6, 7]. This association has been widely reported in clinical studies, showing that menopause is associated with an increased risk of impaired glucose tolerance, elevated blood pressure, and elevated triglycerides [8–10]. In animal models, ovariectomy resulted in increased gluconeogenesis and decreased glycogen levels in the liver, uterus, skeletal muscle, and myocardium [11]. However, the molecular connection between insulin resistance and estrogen deficiency has been attributed to GLUT4, the primary glucose transporter responsible for insulin-stimulated glucose uptake in muscle and adipocytes [12]. In ER*α* knockout mice (ERKO), GLUT4 translocation to the membrane is altered, and expression is significantly reduced [13]. These metabolic irregularities manifest as dysfunction of adenosine monophosphate-activated protein kinase (AMPK), which leads to alterations in the components of the insulin signaling pathway. These components include the insulin receptor, insulin receptor substrate-1 (IRS-1), phosphatidylinositol 3-kinase (PI3K), and protein kinase B (Akt) [14, 15]. Kim et al. demonstrated that 17*β*-estradiol can simultaneously activate IRS-1/Akt and AMPK in 3T3-L1 adipocytes, even in the absence of insulin, which influences the expression of genes related to glucose metabolism [15].

Epidemiologic studies have shown that individuals with borderline-impaired glucose tolerance tend to have stiffer arteries than those with normal glucose tolerance [16–18]. The relationship between insulin resistance and vascular stiffness has been attributed to a decrease in NO production [17]. Physiological levels of insulin promote NO production, through insulin-like growth factor-1 (IGF-1) PI3K/Akt-related pathways to promote eNOS activity in endothelial cells [19], whereas insulin resistance induces endothelial dysfunction in mesenteric vessels, characterized by impaired PI3K and enhanced mitogen-activated protein kinase- (MAPK-) dependent ET-1 secretion [20]. The impairment of NO production dependent on insulin signaling has been linked to angiotensin II (Ang II) [21, 22]. Ang II inhibits the insulin-induced activation of the PI3K pathway, leading to a reduction in endothelial nitric oxide (NO) production and Glut-4 translocation in insulin-sensitive tissues, resulting in insulin resistance [22, 23].

However, the decrease in NO levels is not the only disturbance involved in endothelial dysfunction. Insulin resistance also causes an imbalance in the activity of vascular relaxing and constricting factors [24], including acetylcholine (Ach) and Ang II. Regarding Ang II, several studies have shown that hypertensive ovariectomized rats exhibit increased Ang II vascular reactivity and overexpression of the AT1 receptor for angiotensin [25–28]. The cardioprotective effects of estrogens have been demonstrated in models of myocardial infarction, where vascular reactivity to Ang II is reduced in females with intact ovaries compared to males [29]. However, little information is available on the effects of ovariectomy in normotensive aged rats. One consequence of the excessive Ang II activity is an increase in calcium sensitivity. This increased sensitivity is a result of the activation of AT1 receptors by Ang II, which leads to an increase in cardiac L-type Ca channel current (ICa) through the stimulation of protein kinase C (PKC) [30]. Furthermore, research has shown that ovariectomy increases ICa sensitivity to depolarization-induced contractions when compared to intact females [31]. Regarding Ach, it has been reported that menopause affects endothelium-dependent vasodilation in normotensive and hypertensive women, whereas endothelium-independent vasodilation remains unaffected [32].

In brief, the pressure overload caused by the hyperreactivity of vasoconstrictor agents and the decrease in vasorelaxant activity, in vascular smooth muscle, increases peripheral resistance, exacerbating the incidence and severity of CVD [33], which is reversed by estrogen receptor agonists [31, 34]. To avoid this, estrogen replacement therapy (ERT) has been chosen. In recent years, accumulating data from randomized controlled trials of hormone replacement therapy (HRT) have revealed different responses depending on the timing of HRT initiation relative to age and time since menopause. Specifically, HRT appears to confer benefits in terms of coronary heart disease (CHD) events and all-cause mortality when initiated in younger women (younger than 60 years of age) soon after menopause (within 10 years), but it appears to have little to no effect, or even possible adverse effects when initiated in older women (older than 60 years of age) far from menopause (more than 20 years) [35–38].

In summary, the current knowledge has demonstrated the importance of hormonal changes, particularly estrogen deficiency, in contributing to insulin resistance and endothelial dysfunction. However, it remains unclear how the timing of estrogen replacement therapy (ERT) initiation affects glucose and insulin balance and their association with the activity of key vasoactive factors such as angiotensin II, KCl, and acetylcholine in aged ovariectomized Wistar rats. While existing studies have demonstrated the importance of ERT timing in cardiovascular health, there is a need for focused investigations in this animal model to provide insight into the potential benefits or risks of ERT on vascular function. Therefore, the present contribution is aimed at evaluating the early and late ERT on glucose and insulin balance and its association with the aortic vascular reactivity to angiotensin II, KCl, and acetylcholine in old ovariectomized Wistar rats.

## 2. Methods

### 2.1. Animals

Eighteen-month-old female Wistar rats were obtained from the animal facility of the Cuautitlan Superior Studies Faculty. Rats had free access to food and water and were housed at 25°C under a 12-hour light-dark cycle. All animal procedures were performed in accordance with the Federal Regulation for Animal Experimentation and Care (SAGARPA, NOM-062-ZOO, 1999, Mexico), the local ethics committee number C19_13 (CICUAE- FESC), and the National Institutes of Health Guide for the Care and Use of Laboratory Animals (NIH Publication No. 8023, revised 1978, USA).

### 2.2. Study Design

Animals were randomized into several groups: (1) sham, (2) 10-week postovariectomy (10 w), (3) 10 w postovariectomy+early estradiol therapy (10 w-early E2), (4) 20-week postovariectomy (20 w), and (5) 20-week postovariectomy+late estradiol therapy (20 w-late E2). Early administration of 17*β*-estradiol (E2) was started three days after ovariectomy (comparable to the situation found in humans two months after menopause [39]), while late therapy was started ten weeks after ovariectomy (similar to the situation in humans five years after menopause [39]). In both groups, E2 was administered daily at a dose of 5 *μ*g/kg/day (being one of the lowest doses reported in the literature [40–43]). The interval between ovariectomy and the initiation of E2 therapy varied in both groups (10 or 20 weeks). This variation was designed to ensure the same duration of treatment (10 weeks), regardless of whether E2 therapy was started, shortly after ovariectomy (3 days) or after a longer interval (10 weeks). Evaluation of vascular function and sampling were performed at 10- or 20-week postovariectomy in all experimental groups, with food deprivation 12 hours prior to the experimental procedure.

### 2.3. Bilateral Ovariectomy

Rats were anesthetized with pentobarbital (35 mg/kg i.p.) and underwent bilateral ovariectomy as previously described [44]. The sham group underwent the same procedure, except for ovary removal. All animals received postoperative analgesia with tramadol (10 mg/kg i.p.) and topical betamethasone.

### 2.4. Serum Glucose Level Quantification

Serum glucose was measured in serum by spectrophotometry at the end of the treatment in all experimental groups. The assay was performed according to the manufacturer's instructions (Wiener Lab, glycemia AA enzyme).

### 2.5. Serum Insulin Level Quantification

The Millipore ELISA kit (RAB0904, Sigma-Aldrich, lot number 0712I0743) was used according to the supplier's instructions.

### 2.6. HOMA-IR

Homeostasis model assessment (HOMA) was used to determine the insulin resistance (HOMA-IR). The HOMA-IR index was calculated using the following formula: HOMA − IR = [(fasting glucose (mmol/L) × fasting insulin (*μ*UI/mL))/22.5] [45].

### 2.7. Serum NO Level Quantification

NO was quantified by the Griess reaction, an indirect method based on the measurement of NO_2_^−^ concentration, since NO_2_^−^ and NO_3_^−^ are the stable products of NO metabolism. VaCl_3_ (0.8% diluted in 10% HCl) was used to reduce NO_3_^−^ to NO_2_^−^ in the serum samples. Proteins in serum samples were precipitated by ethanol : water solution (7 : 1) by adding 100 *μ*L of serum and 200 *μ*L of the solution, followed by centrifugation at 14,500 rpm for 20 min. In a microtiter plate, we add 100 *μ*L of the supernatant sample, 100 *μ*L of VaCl_3_, 50 *μ*L of sulfanilamide (2% diluted in 10% HCl), and 50 *μ*L of N-(1-Naphthyl)ethylenediamine (0.2% diluted in 10% HCl). After 20 min incubation at room temperature, the absorbance was read at 540 nm using an ELISA reader. NO concentrations in samples were determined from a linear sodium nitrite standard curve constructed from 0 to 230 *μ*M [46].

### 2.8. Assessment of Vascular Reactivity

The thoracic aorta was dissected and placed in fresh Krebs-Henseleit solution containing 118 mM NaCl, 4.7 mM KCl, 1.2 mM KH_2_PO_4_, 1.2 mM MgSO_4_-7H_2_O, 2.5 mM CaCl_2_-2H_2_O, 25 mM NaHCO_3_, 11.7 mM dextrose, and 0.026 mM calcium disodium EDTA and continuously oxygenated with a 95% O_2_/5% CO_2_ mixture. The adipose, connective, and adherent tissues of the aorta were removed. They were cut into rings (3 mm in length). Each ring was transferred to 10 mL isolated tissue chambers containing the Krebs-Henseleit solution at 37°C and pH 7.4 and continuously aerated with a mixture of 95% O_2_/5% CO_2_. To record semi-isometric force development, the rings were suspended between two wire hooks (Nubryte wire), one attached to the bottom of the chamber and the other to a force transducer (BIOPAC TSD125C) connected to a BIOPAC MP100A-CE system (BIOPAC Systems Inc., Santa Barbara, CA, USA) using AcqKnowledge software 3.8.1. The initial tension applied to each aortic annulus was set at 3 g. Endothelial integrity was assessed in aortic rings treated with 10^−6^ M phenylephrine (Phe) by the addition of 10^−5^ M ACh, and a 60% vasodilator response was defined for the inclusion of the aortic ring in the experiment. The vasoconstrictor effect of Ang II was tested in a concentration range from 10^−12^ to 10^−6^ M, concentration-response curves were also made for KCl (10–60 mM), and finally, the vasodilator effect was tested at 10^–12^-10^−6^ M ACh.

### 2.9. Measurements of Blood Pressure and Heart Rate

The study utilized a noninvasive tail-cuff device to record systolic (SBP), diastolic (DBP), and mean (MBP) blood pressure in rats. To reduce animal stress and ensure consistency, the rats were trained for a week in a plastic restrainer. BP was recorded using an SPAM equipment (INC-ICh, Mexico), and Sievart 1 software was utilized for data analysis. MBP is calculated as follows: MBP = diastolic BP + 1/3 (systolic BP − diastolic BP) [47].

### 2.10. Statistical Analysis

Our analysis was conducted using one-way analysis of variance (ANOVA) followed by the Student-Newman-Keuls post hoc test to compare groups. We present all data as mean standard error (SEM). Concentration-all data are presented as mean standard error (SEM). Concentration-response curves for KCl, ACh, and Ang II (*n* = 10) were expressed using the aortic contraction in g ± SEM. Two-way ANOVA and Sidak's post hoc test were utilized to evaluate variations in data, comparing against the control group (^∗^) and between groups. A *P* value of less than 0.05 revealed statistical significance.

## 3. Results

### 3.1. Ovariectomy Leads to an Imbalance of Glucose and Insulin, Resulting in an Increase of the HOMA-IR Index

**Figure 1:**
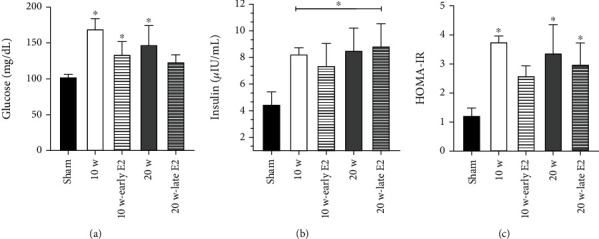
Ovariectomy promotes glucose/insulin imbalance. (a) Glucose serum levels. 10 w, 10 w-early E2, and 20 w groups showed an increase in glucose blood levels vs. the sham group (^∗^*P* = 0.028, 0.032, and 0.045, respectively). (b) Insulin serum levels. All groups (10 w, 10 w-early E2, 20 w, and 20 w-late E2) presented higher insulin levels vs. the sham group (^∗^*P* = 0.016, 0.024, 0.004, and <0.001, respectively). (c) HOMA-IR index. 10 w, 20 w, and 20 w-late E2 groups showed insulin resistance vs. the sham group (^∗^*P* = 0.002, 0.010, and 0.018, respectively). Results are shown as the mean ± SEM (*n* = 10). One-way analysis of variance (ANOVA) followed by the Student-Newman-Keuls post hoc test was used for comparisons between groups.

First, blood glucose concentration was measured at the end of treatment in all groups, as shown in Figure 1(a). The results indicated a significant increase in glucose levels in all the groups compared to the sham group (10 w: *P* = 0.028, 10 w-early E2: *P* = 0.032, and 20 w: 0.045), except for the 20 w-late E2 group.

When insulin was measured (Figure 1(b)), all groups showed an increase compared to the sham group (10 w: *P* = 0.016, 10 w-early E2: *P* = 0.024, 20 w: 0.004, and 20 w-late E2: *P* < 0.001). No significant differences in insulin levels were noted between the ovariectomy groups with and without E2 therapy. Finally, to evaluate insulin resistance, we utilized the HOMA-IR (homeostasis model assessment of insulin resistance) index and observed a significant increase in the 10 w group (*P* = 0.002), 20 w group (*P* = 0.010), and 20 w-late E2 group (*P* = 0.018) compared to the sham group. However, we found no significant difference between the 10 w-early E2 group and sham group (Figure 1(c)).

### 3.2. Early E2 Therapy Increases NO Serum Levels at 10 w Postovariectomy

**Figure 2:**
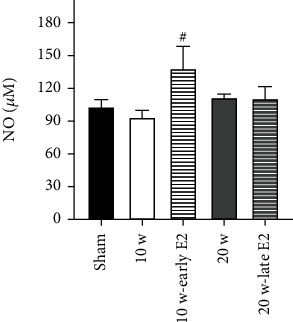
Early E2 therapy increases NO serum levels at 10 w postovariectomy. No significant differences in NO serum levels were found in all groups vs. the sham group. However, the 10 w-early E2 group increases NO serum concentrations vs. the 10 w group (^#^*P* = 0.045). NO: nitric oxide. Results are presented as the mean ± SEM (*n* = 10). One-way analysis of variance (ANOVA) on ranks followed by the Student-Newman-Keuls post hoc test was used for comparisons between groups.

Additionally, serum NO concentrations were evaluated to determine whether ovariectomy and E2 therapy modified this biomarker (Figure 2). The results revealed no significant differences between the ovariectomized groups compared to the sham group. However, the 10 w-early E2 group exhibited higher NO levels compared to the 10 w group (^#^*P* = 0.045).

### 3.3. E2 Therapy Mitigates the Ovariectomy-Induced Increase in Vascular Contraction to Ang II

**Figure 3:**
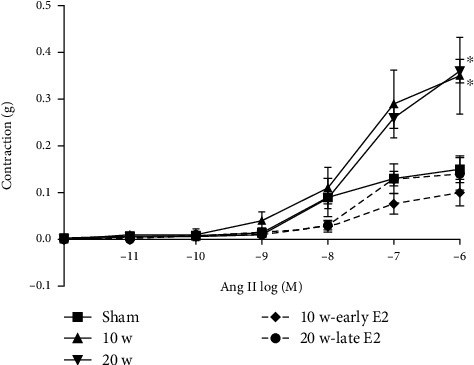
Early and late E2 therapy decreases the vascular contraction to Ang II. In vitro vascular response to Ang II in aortic rings. 10 w and 20 w increase vascular contraction vs. the sham group (^∗^*P* < 0.0001 and ^∗^*P* < 0.0001, respectively). Values are the mean ± SEM. Two-way ANOVA and Sidak's post hoc test were used to assess differences in the data.

To investigate vascular function, we conducted concentration-response for KCl, acetylcholine (Ach), and angiotensin II (Ang II). We observed modifications in the EC50 of all three substances (Table 1). Ovariectomy resulted in a significant increase in contraction (*P* < 0.0001) after 10 and 20 weeks of surgery (Figure 3); hence, it appears that estrogens enhance the affinity of Ang II for the AT_1_ receptor that seems to be ameliorated by estrogens (Table 1). The E2 therapy restored the response to Ang II to EC50 values comparable to the sham group (10 w-early E2 = 5.37 × 10^−8^ M and 20 w-late E2 = 7.76 × 10^−8^ M).

### 3.4. Vascular Response to KCl and Acetylcholine Is Increased 10 Weeks after Ovariectomy

**Figure 4:**
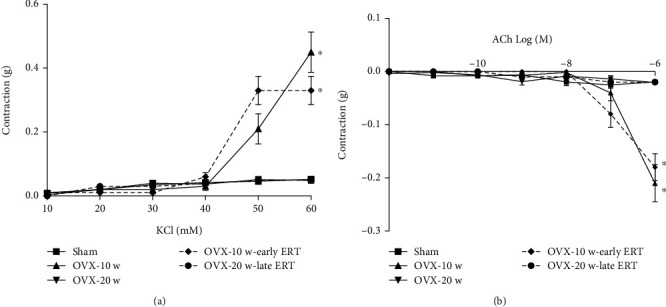
Vascular response to KCl and acetylcholine increases at 10-week postovariectomy. (a) In vitro vascular response to KCl in aortic rings. 10 w and 10 w-early E2 increase aortic contraction vs. the Sham group (^∗^*P* < 0.0001 and ^∗^*P* < 0.0001, respectively). (b) In vitro vascular response to Ach in aortic rings. 10 w and 10 w-early E2 showed an increase in vascular relaxation response to Ach vs. the sham group (^∗^*P* < 0.0001 and ^∗^*P* < 0.0001, respectively). KCl: potassium chloride; Ach: acetylcholine. Values are the mean ± SEM. Two-way ANOVA and Sidak's post hoc test were used to assess differences in the data.

To test the voltage-gated Ca^+2^ channel response in aortic rings, high concentrations of KCl were added [48] (Figure 4(a)). An increase in the contraction and EC50 was observed in the 10 w group (*P* < 0.0001) and in the 10 w-early E2 group (*P* < 0.0001) in comparison to the sham group (EC50 = 2.63 × 10^−2^ M in sham group vs. 4.46 × 10^−2^ M in 10 w group and 5.49 × 10^−2^ M in 10 w-early E2). This response was restored after 20-week postovariectomy with and without E2 treatment. However, only the 20 w-late E2 group exhibited a decrease in EC50 and PD2 (1.65 × 10^−2^ M, 1.78) versus the sham group (2.63 × 10^−2^ M, 1.58).

The ACh EC50 was found to increase in both groups at 10-week postovariectomy with and without estrogen therapy (*P* < 0.0001) (1.99 × 10^−6^ and 3.38 × 10^−4^ vs. 4.36 × 10^−10^ sham). However, no significant changes were observed at 20-week postovariectomy (Figure 4(b)).

### 3.5. Blood Pressure and Heart Rate Changes Depend on Postovariectomy Time

Finally, the blood pressure (BP) and heart rate (HR) of all the experimental groups were measured (Table 2). A significant increase in diastolic blood pressure (DBP) was observed in groups with 20-week postovariectomy (*P* = 0.038), with and without E2 therapy (82 ± 1.3 and 83 ± 1 mmHg, respectively). Although no significant differences in the other parameters were found, the mean blood pressure (MBP) seems to be lower in the 10 w and 10 w-early E2 groups vs. the sham group. Systolic blood pressure (SBP) tends to decrease in the 10 w group and the 10 w-early E2 group (112 ± 5 and 109 ± 3 mmHg, respectively) in comparison with the sham group (119 ± 2). Additionally, HR tends to decrease in all groups compared to sham, except for the 20 w-late E2 group.

## 4. Discussion

The results of our contribution showed that the arterial blood pressure and vascular response to KCl and acetylcholine are dependent on postovariectomy time, while the HOMA-IR and vascular response to Ang II are modified by the E2 therapy. Hormone depletion resulting from ovariectomy has been associated to metabolic imbalances and altered vascular reactivity [49–51]. However, it remains unclear how the changes occur over time following ovariectomy, as well as the impact of early or late administration of E2 therapy on aged females. Our work reveals that certain physiological biomarkers may vary depending on the postovariectomy time. In agreement with our results, some clinical studies have indicated that cardiovascular health changes are influenced by the menopause transition beyond aging, emphasizing the importance of studying this process from a temporal perspective [36].

In our study, the HOMA-IR index indicated an imbalance in glucose metabolism following ovariectomy in both the 10 w and 20 w groups. This may be due to depletion of ovarian hormones, as previous research has linked a decrease in circulating estrogens with impaired glycemic control in females, resulting in insulin resistance (IR) [52]. Additionally, it has been demonstrated that loss of ovarian hormones can also led to central obesity and dyslipidemia, ultimately increasing the risk for metabolic syndrome [53]. Ahmed et al. demonstrated that estrogen deficiency results in an imbalance between the ER*α* and ER*β* receptors and decreases the expression of ER*α* [54]. This decrease in the ER*α* has been linked to IR through a decrease in GLUT4 expression and translocation [13, 55]. Our study indicates that early E2 administration can prevent an increase in the HOMA-IR index. In agreement with our results, Kim et al. demonstrated that 17*β*-estradiol activates IRS-1/Akt and AMPK, improving glucose metabolism even without insulin [15]. Moreover, E2 prevents apoptosis in pancreatic *β* cells through genomic and nongenomic pathways and reduces oxidative stress at physiological doses [56, 57]. Additionally, clinical studies reveal that E2 therapy reduces IR in diabetic women when administered at perimenopause [58]. However, late E2 treatment had a different effect, leading to an increase in the HOMA-IR index. Our findings align with Pereira et al., who showed that E2 treatment resulted in an increase in insulin-stimulated glucose disposal (GDR) among early postmenopausal women but a decrease among late postmenopausal women [59]. Therefore, the effect of E2 treatment on glucoregulatory insulin action is influenced by the time elapsed between menopause and the initiation of therapy, which may be attributed to changes in estrogen receptor expression, favoring glucose homeostasis with ER*α* while having a detrimental effect with ER*β* [59]. Our results suggest a transition from beneficial to detrimental effects with prolonged estrogen deficiency. Additionally, the mechanism by which E2 confers cardioprotection against IR in women has not been fully explained [60]. Consequently, it is important to note that there is a lack of temporal studies regarding the time after ovariectomy and the start of E2 therapy. In this sense, some clinical researchers have shown a strong association between the metabolic syndrome (which includes IR) and the menopausal transition, with the severity of this syndrome increasing during the late perimenopausal years rather than in the postmenopausal period [36].

In this context, metabolic diseases characterized by IR produce endothelial dysfunction, leading to the development of hypertension [61, 62]. The main cause of vascular dysfunction due to IR has been attributed to the decrease in NO bioavailability [63–65]. Normal insulin levels stimulate NO production through IGF-1 and PI3K/Akt pathways, thereby increasing eNOS activity in endothelial cells [19]. In contrast, IR leads to endothelial dysfunction with impaired PI3K and increased MAPK-related endothelin 1 secretion [20]. Decreased NO contributes to high blood flow and shear stress, which causes cell damage [66] and accelerates the synthesis of advanced glycosylation end products (AGE) [64, 65]. In this study, we found that early E2 therapy increased NO levels compared to the OVX age-matched group (10 w). Consistent with our findings, estrogens have been shown to increase NO levels in vascular smooth cells as a cardioprotective mechanism [67]. Estrogens and GPER-selective agonists cause the phosphorylation of eNOS at Ser1177 and induce NO production through GPER-dependent pathways, involving c-Src, EGFR, PI3K, and ERK signaling [68]. With respect to late E2 therapy, no changes were found, confirming that E2 therapy has time-dependent effects on NO production.

IR affects not only the final metabolites that promote vasorelaxation but also the ability of the vascular smooth muscle to respond to a stimulus [69]. Ovariectomized groups without E2 therapy showed an increase in vascular reactivity to Ang II as well as insulin resistance. This association has been studied in males, where Ang II was found to increase phosphorylation of IRS-1 and inhibit insulin-stimulated activation of eNOS, suggesting that Ang II may be the link between insulin resistance and the development of vascular cell dysfunction [70]. Also in males, Ang II treatment produces a lower response to insulin with reduced rates of glucose oxidation in the heart [71]. Our study showed that E2 therapy, both early and late, attenuated aortic contraction to Ang II, counteracting the increased response resulting from ovariectomy. In agreement with our results, the increase in vascular response to Ang II due to ovariectomy was related to the increase in AT1 expression and angiotensin converting enzyme (ACE) activity at 5 weeks after ovariectomy, which can be prevented by the administration of low-dose estradiol but not by high-dose estradiol [72]. In addition, Xu et al. demonstrated that ovariectomy causes a decrease in Ang II and atrial natriuretic peptide (ANP) while increasing renin activity, but these effects can be prevented by estrogen therapy [73].

Excessive Ang II activity contributes to increased calcium sensitivity, primarily through AT1 receptor activation, resulting in increased cardiac L-type Ca channel current (ICa) via protein kinase C (PKC) stimulation [30]. Furthermore, calcium regulation is one of the most affected processes after long-term ovariectomy. In the heart, ovariectomy causes a decrease in myofilament Ca^2+^ sensitivity and an increase in spontaneous Ca^2+^ release in aged females [74, 75]. Therefore, to study vascular Ca^2+^ channel reactivity, we performed concentration-response curves to KCl, which induces the opening of voltage-operated Ca^2+^ channels (VOCCs) [76] and activates Ca^2+^-dependent myosin light chain kinase (MLCK) [77]. Our results showed an increase in Ca^+2^ channel reactivity in aortic rings at 10- but not 20-week postovariectomy. In agreement with our results, Valencia-Hernández et al. demonstrated that Ca^2+^ vascular reactivity in ovariectomized female rats depends on the postovariectomy time, finding an increase in Ca^2+^ response at 8 weeks, but not at 1 or 3 weeks after the surgery [78]. Kim and Yang also found an increase in K_Ca_ channel conductance two weeks after ovariectomy [79]. In this sense, some studies have shown that ovariectomy leads to an increase in Ca^2+^ L-type channel activity, which increases Ca^2+^ influx [75, 80]. Regarding E2 therapy, our results showed that potassium-activated calcium channels do not change their reactivity due to the presence or absence of E2. This result may be related to the dose that was used since Belfort et al. found that E2 has vasodilator effects on KCl-precontracted isolated arteries from women, only at high estradiol concentrations [81]. In addition, in vitro studies confirm that only high estrogen concentrations are able to inhibit voltage-dependent Ca^2+^ channels in coronary arterial smooth muscle cells [75, 82].

In this study, we also evaluated the response to acetylcholine because this neurotransmitter is one of the most important substances that regulate vascular tone. ACh activity has been linked to estrogens by Andersen et al., who showed that long-term E2 treatment (7 days) significantly increased aortic ring vasodilatation in response to acetylcholine [67]. This supports our results in the 10 w-early E2, where this therapy appears to enhance ACh-induced vasorelaxation.

However, the increase in vascular response to ACh in the 10 w group may have another explanation. As we have discussed, the 10 w and 10 w-early E2 groups showed an increase in Ca^+2^ L-type channel reactivity, as well as in vascular response to ACh. Thus, we propose that ACh may induce a more pronounced vasorelaxant response in the 10 w group to compensate for the calcium influx. In agreement with our results, Martorell et al. demonstrated that after ovariectomy, there is an increase in ACh vascular response through cyclooxygenase-2 (COX-2) overexpression, which stimulates eNOS activity leading to overproduction of NO. Therefore, the increase in ACh vascular response at 10-week postovariectomy could be explained by an inflammatory process activated by COX-2 leading to an increase in vascular NO [83]. In addition, Yasuda et al. showed that ovariectomy increases the expression of muscarinic acetylcholine receptor 3 (M3) [84], which is present in endothelial cells and smooth muscle cells [85] and is responsible for the vasodilation mediated by the increase in eNOS activity.

Finally, it is known that the prevalence of hypertension increases after menopause [86]. Menopausal vascular dysfunction has been associated with increases in aortic stiffness, Ang II and endothelin activity, and peripheral resistance, resulting in increased BP [87]. Despite the increased vascular response to Ang II, we found an increase in DBP only 20 weeks after ovariectomy with or without E2 therapy. Thus, the increase in DBP may represent the beginning of the development of hypertension in this model. In agreement with our results, some studies have shown that ovariectomy increases BP in a time-dependent manner [73], which could explain why there is no change at 10-week postovariectomy. Nevertheless, it is necessary to perform more studies at different times after ovariectomy to better determine the evolution of physiological and pathological changes after menopause.

## 5. Conclusion

Our study highlights the complex interplay between hormonal changes and vascular health in a menopausal model. Ovariectomy without E2 therapy resulted in increased vascular reactivity to Ang II, accompanied by insulin resistance. Notably, IR emerged as a key player, with early estrogen therapy mitigating these consequences of ovariectomy, possibly through enhanced nitric oxide production, thereby preserving vascular function. However, arterial blood pressure and vascular responses to KCl and acetylcholine showed significant time-dependent variations after ovariectomy, emphasizing the importance of considering the postovariectomy time frame and the initiation of E2 in understanding the physiological changes that occur during menopause.

## Figures and Tables

**Table 1:** EC50 (M) and PD2 (-log M) values to KCl, ACh, and Ang II.

Group	KCL		ACh		Ang II	
	EC50 (M)	PD2	EC50 (M)	PD2	EC50 (M)	PD2
Sham	2.63 × 10^−2^	1.58	4.36 × 10^−10^	9.36	4.28 × 10^−8^	7.37
10 w	4.46 × 10^−2^∗^^	1.35^∗^	1.99 × 10^−6^∗^^	5.70^∗^	6.76 × 10^−8^	7.17
20 w	2.81 × 10^−2^	1.55	4.36 × 10^−9^	8.36	1.28 × 10^−7^∗^^	6.89^∗^
10 w-early E2	5.49 × 10^−2^∗^^	1.26^∗^	3.38 × 10^−4^∗^^	3.47^∗^	5.37 × 10^−8^	7.27
20 w-late E2	1.65 × 10^−2^∗^^	1.78	1.77 × 10^−9^	8.75	7.76 × 10^−8^	7.11

Effective concentration 50 (M). PD2: -log M. These results are the mean of 10 experiments. One-way ANOVA followed by a Student-Newman Keuls post hoc test. ^∗^*P* < 0.05 vs. sham.

**Table 2:** Blood pressure and heart rate changes depend on postovariectomy time.

Parameter	Sham	10 w	10 w-early E2	20 w	20 w-late E2
SBP (mmHg)	119 ± 2	111 ± 5	109 ± 3	122 ± 2	116 ± 2
DBP (mmHg)	77 ± 1	79 ± 2	75 ± 1	83 ± 1^∗^	82 ± 1^∗^
MBP (mmHg)	91 ± 1	89 ± 3	86 ± 2	95 ± 1	94 ± 1
HR (beats/min)	372 ± 7	349 ± 9	354 ± 7	356 ± 11	384 ± 6

DBP increases at 20 w postovariectomy with and without ERT vs. sham (^∗^*P* = 0.038). MBP: mean blood pressure; SBP: systolic blood pressure; DBP: diastolic blood pressure; HR: heart rate. The results are the mean ± SEM (*n* = 10). One-way analysis of variance (ANOVA) followed by the Student-Newman-Keuls post hoc test was used for comparisons between groups.

## Data Availability

The data used to support the findings of this study are included within the article.
